# A Systematic Evaluation of Curcumin Concentrations and Blue Light Parameters towards Antimicrobial Photodynamic Therapy against Cariogenic Microorganisms

**DOI:** 10.3390/pharmaceutics15122707

**Published:** 2023-11-30

**Authors:** Patricia Comeau, Adriana Manso

**Affiliations:** 1 Department of Oral Health Science, The University of British Columbia, Vancouver, BC V6T 1Z3, Canada; patricia.comeau@concordia.ca; 2 Department of Chemical and Materials Engineering, Concordia University, Montreal, QC H3G 2W1, Canada

**Keywords:** curcumin, photosensitizer, blue light, photodynamic therapy, antimicrobial, dental caries, *Streptococcus mutans*, *Candida albicans*, reactive oxygen species, light absorption

## Abstract

Dental caries is a highly preventable and costly disease. Unfortunately, the current management strategies are inadequate at reducing the incidence and new minimally invasive strategies are needed. In this study, a systematic evaluation of specific light parameters and aqueous curcumin concentrations for antimicrobial photodynamic therapy (aPDT) was conducted. Aqueous solutions of curcumin were first prepared and evaluated for their light absorbance after applying different ~56 mW/cm^2^ blue light treatments in a continuous application mode. Next, these same light treatments as well as different application modes were applied to the curcumin solutions and the molar absorptivity coefficient, reactive oxygen species (ROS) release, minimum inhibitory concentration (MIC), and minimum bactericidal concentration (MBC) for *Streptococcus mutans* and the MIC and minimum fungicidal concentration (MFC) for *Candida albicans* were measured. After up to 1 min of light treatment, the molar absorptivity of curcumin when added to culture media was lower than that for water only; however, at higher energy levels, this difference was not apparent. There was a noted dependence on both ROS type and cariogenic microorganism species on the sensitivity to both blue light treatment and application mode. In conclusion, this study provides new information towards improving the agonistic potential of aPDT associated with curcumin against cariogenic microorganisms.

## 1. Introduction

Good general health and well-being are directly linked to a healthy mouth. However, over the last 30 years, the global case numbers for oral diseases have increased by 1 billion [1]. In 2019 alone, oral diseases were the most widespread condition with more than 3.5 billion people affected worldwide and roughly 2 billion of these cases were attributed to untreated caries on permanent teeth [1]. Oral diseases such as dental caries are multifactorial and largely preventable, benefiting from early diagnosis and treatment to achieve proper management. Unfortunately, owing to challenges such as socioeconomic inequalities, dental caries persists with an unparalleled burden which is particularly relevant in disadvantaged and marginalized populations, thus remaining a highly prevalent worldwide health concern [1,2].

The WHO 2022 report defines dental caries as the gradual loss and decay of hard dental tissue as a result of the conversion of free sugars into acids by bacteria [1]. This disease is a consequence of interactions on teeth between microorganisms, microbial products, host saliva, and diet (i.e., sugar), and results in the formation of pathogenic biofilm and eventually tooth decay [3]. *Streptoccocus mutans* (*S. mutans*), as one such cariogenic bacteria, can quickly orchestrate the production of pathogenic biofilms when the dental surface is frequently exposed to dietary sucrose [4]. Meanwhile, *S. mutans*-derived enzymes, such as glucosyltransferases, utilize sucrose to produce exopolysaccharides (EPS), which is a necessary component of cariogenic biofilms [4]. Acidogenic/aciduric microorganisms, including *S. mutans*, produce acidic microenvironments within the biofilm due to their metabolism of sugars. This environment then further aids in the selective growth of these microorganisms and promotes continuous dissolution of tooth enamel [4]. It has been shown that *Candida albicans* (*C. albicans*) enhances not only the biofilm assembly, but also supports *S. mutans* viability within the EPS matrix [4,5,6]. Furthermore, *C. albicans* has been shown to contain a number of proteolytic enzymes capable of aiding collagenolysis [6,7,8], which is seen with dental caries. Both *S. mutans* and *C. albicans* are thus two oral species commonly investigated in dual-species models due to their synergisms in disease progression [3]. As a result, in the development of material-based approaches for dental caries management, these cells in the dual-species biofilm model are of great relevance.

The prevalence of dental caries should be effectively decreased by reducing sugar consumption and utilizing proper preventative strategies. For example, fluorides have been extensively studied for more than 70 years as one such preventive strategy and may be self-applied as toothpaste and mouth rinses, professionally applied as varnish or gels, and in community-based systems through the fluoridation of water, salt, or milk [1]. Additionally, the cost associated with regular visits to dental care professionals to allow access to the most effective preventive alternatives, such as high fluoride content oral care products (e.g., rinses, gels, and varnishes), is also a limiting factor for some populations. In parallel, it is important to consider that even low fluoride intake may lead to a detectable level of fluorosis [9]. As a result, there has been a significant shift to developing more accessible and natural management strategies, including both preventatives and therapeutics. While there are several antimicrobial agents that have been proven to be clinically effective against cariogenic microorganisms, including chlorhexidine rinse, such treatments are hindered by side effects including bitter taste, teeth staining, the non-selective nature of the treatment, allergic reactions, and some risk of developing antimicrobial resistance [10]. As a non-invasive, repeatable, and targeted method, antimicrobial photodynamic therapy (aPDT) has become a powerful antimicrobial alternative in dental practice for managing the oral cavity microbiota and in general medicine for combating antibiotic-resistant bacteria [10,11,12].

Photodynamic therapy (PDT) is a non-thermal photochemical reaction that consists of three main components: a photosensitizer (PS), oxygen, and light [10,13,14]. Upon administering light of a matching wavelength, the PS becomes activated and reactive oxygen species (ROS) are produced, leading to permanent damage of the targeted cariogenic cells [10]. This photodynamic inactivation of the bacteria (named antimicrobial PDT or “aPDT”) is believed to result from the passage of the PS across the cytoplasmic membrane and is critical for inducing irreversible damage to the bacterial cell [15]. An ideal PS should be a pure substance which is stable at room temperature, activated upon selective light application, and presents with optimal pharmacokinetic properties [13]. The PS would also ideally have preferential affinity for binding to microorganism cells, have a broad spectrum of antimicrobial action, and need a small drug-light interval [13]. While there are several PSs that have been of interest for aPDT in dentistry, curcumin has garnered significant attention in this field owing in part to its historical use in other medical fields, and its compatible properties for use in aPDT [16]. Curcumin presents some important advantages, including having a maximum absorption peak in the blue light wavelengths (i.e., 420–450 nm), its low cost, and its effectiveness at targeting several different pathogenic microorganisms, including *S. mutans* and *C. albicans* [16,17,18,19,20,21,22,23,24,25,26]. Curcumin is a dye obtained from the root of *Curcuma longa* and while considered soluble in ethanol and DMSO, it is relatively insoluble in water [27,28]. A notable challenge to comparing the aPDT literature on the same PS is the minimal consistency in study parameters and, as a result, the measured microorganism responses vary significantly. In addition, it is important to highlight that there has been considerable effort in the aPDT literature to investigate the performance of a PS dissolved in certain solvents (e.g., ethanol, DMSO) [29], which are themselves somewhat cytotoxic to the targeted cells. For its future use in dental materials for oral care, it is important to understand the action of curcumin within an aqueous environment, including in the presence of culture media when benchtop investigations are performed.

In addition, while there are a few different light source options, the quasi-monochromatic nature and low cost of light emitting diode (LED) have led to its widespread use in dentistry and in aPDT in general [13]. Blue LED devices are readily available in most dental offices and in illuminated trays for home use. Thus, the adoption of an aPDT approach linked to blue light should occur relatively quickly. Based on early investigations of other PSs, the efficacy of aPDT has been shown to be dependent on several light-based factors, including the light wavelength and its overlap with the photosensitizer’s absorption peak, the output power, the irradiation duration, and the light source’s operational mode (continuous or fractional) [30,31,32]. However, while several studies have investigated the response of cariogenic microorganisms to curcumin in dark and light-treated conditions towards PDT approaches [16,27,29], no study has systematically investigated these responses as a function of curcumin concentration, blue light energy, light operation mode, and their synergism. Furthermore, while the impact of ROS on microorganisms has been previously studied [33], it is rare to see the produced ROS quantified in any cell-free aPDT investigation of curcumin.

To address these gaps in knowledge, this study proposes four general hypotheses. First, that there is a dependence of curcumin absorbance of blue light on the culture media type; second, that the light energy applied does impact the minimum inhibitory concentration (MIC), minimum bactericidal concentration (MBC), or minimum fungicidal concentration (MFC); third, that the mode of light application does affect the aPDT response; and fourth, that there is an increase in ROS production as a result of increased light energy and curcumin concentration.

## 2. Materials and Methods

### 2.1. Characterization of Curcumin Powder

Curcumin powder (>95% purity, Santa Cruz Biotechnology, Dallas, TX, USA) was first imaged with a Helios NanoLab 650 Focused Ion Beam Scanning Electron Microscope (FIB-SEM; FEI, Hillsboro, OR, USA). Images were collected under high vacuum, with an accelerating voltage of 1.00 kV and with a working distance of 4.5 mm. Attenuated Total Reflectance–Fourier Transform Infrared (ATR-FTIR) spectra of the powder were also acquired using a Perkin Elmer Spectrum Two FTIR instrument (Perkin Elmer, Waltham, MA, USA) equipped with a Universal ATR sampling accessory consisting of a diamond crystal. The spectral region between 4000 and 500 cm^−1^ was analyzed with 64 scans and a 4 cm^−1^ resolution.

### 2.2. Molar Absorptivity Coefficients of Aqueous Curcumin Solutions

Aqueous solutions of curcumin were prepared in sterile ultrapure water starting at 1250 µg/mL and serially diluted by a factor of two down to 1.22 µg/mL. These solutions were further diluted by a factor of two using three different media: (1) sterile water, (2) BD BBL^TM^ brain heart infusion broth (BHI; BD Canada, Mississauga, ON, Canada), and (3) BD Bacto^TM^ tryptic soy broth (TSB; BD Canada, Mississauga, ON, Canada). The absorbance spectra of each of these solutions were then collected from 350 to 600 nm with a 10 nm step size using an Epoch microplate reader spectrophotometer (BioTek Instruments, Winooski, VT, USA) in a 96-well plate (Costar^®^, Corning Inc., Corning, NY, USA) (n = 5). All media without curcumin (i.e., 0 µg/mL) served as study controls and their absorbances were subtracted from those of the curcumin solutions. A full spectrum was run for each condition without light treatment. In addition, the prepared solutions of curcumin were also read for OD420nm after being light treated with our customized, in-house light device “BioLight” [34] (see Table 1), which was set up with royal blue LEDs (440–460 nm) operating at ~56 mW/cm^2^. Note that this irradiation power was calculated assuming non-collimated light and a working distance set as the distance between the LEDs and the bottom of a well in the 96-well plate [30].

Here, untreated and dark samples served as experimental controls. The molar absorptivity (ε) of curcumin as a function of medium type and light treatment was then calculated using the Beer–Lambert equation (Equation (1)):

A = εlc(1)

where A is the absorbance of the solution at 420 nm, ε is the molar absorptivity (1/(M*cm)), l is the pathlength (0.58 cm for Corning Costar^®^ 96-well flat-bottom plates), and c is the solution concentration (M). Upon plotting the absorbance as a function of curcumin concentration, the slope of the line-of-best-fit is equivalent to εl and from this, the molar absorptivity, ε, was calculated (n = 5).

### 2.3. Quantifying ROS Release Following aPDT

Singlet oxygen and superoxide anions produced during aPDT of aqueous curcumin was quantified according to a previously established colourimetric protocol by Igarashi et al. (2007) [35].

#### 2.3.1. Singlet Oxygen

Singlet oxygen was measured by spectrophotometrically quantifying the bleaching of p-nitrosodimethylaniline (RNO) at 440 nm using imidazole as a selective acceptor of singlet oxygen. First a 20 mM solution of sodium phosphate buffer (NaPB) was prepared by adding the requisite amounts of sodium phosphate monobasic monohydrate and sodium phosphate dibasic heptahydrate powder to ultrapure water. Next, a solution consisting of 50 µM RNO and 50 µM imidazole in 20 mM NaPB was first prepared in an amber bottle to aid in singlet oxygen detection (labeled “solution A”). A stock mixture of curcumin was then prepared at a concentration of 1250 µg/mL in solution A and allowed to mix on a magnetic stirring plate for ~4 h at room temperature. Then, the curcumin solution was serially diluted by a factor of two with solution A in a 96-well plate (highest concentration kept at 1250 µg/mL) down to 1.22 µg/mL, and 0 µg/mL solution A was used as a study control. Each mixture was then diluted by a further factor of two using a 1:1 *v*/*v* acetonitrile/water solution. The optical density at 440 nm was then analyzed using the Epoch plate reader for each plate (OD_440_0_) [35]. Once this dark condition was measured, the plate was light treated according to one of the five treatments provided in Table 1. After each continuous light treatment (1, 5, 15 min) or fractional light segment (1 min, +4 min, +10 min), the plate was stored under aluminum foil for 5 min before reading OD_440_. As curcumin has considerable absorbance at 440 nm, for this assay, a solution of curcumin in water was similarly prepared to that in solution A, and its OD_440_ subtracted from that of the curcumin in solution A (OD_440_t_). The percentage decrease in OD_440_ (Equation (2)) corresponded to the amount of singlet oxygen produced (n = 9).

% OD_440_ Decrease = [OD_440_0_ − OD_440_t_]/OD_440_0_ × 100% (2)

#### 2.3.2. Superoxide Anions

Superoxide anions were quantified by spectrophotometrically measuring the reduction of nitroblue tetrazolium (NBT) as evidenced by an increase in absorbance at 560 nm. A solution consisting of 200 µM NBT in 20 mM NaPB was first prepared in an amber bottle for superoxide anion detection (labeled “solution B”). A stock mixture of curcumin was then prepared at 1250 µg/mL in solution B and allowed to mix on a magnetic stirring plate for ~4 h. After this time, the curcumin solution was serially diluted by a factor of two with solution B in a 96-well plate (highest concentration kept at 1250 µg/mL) down to 1.22 µg/mL. A 0 µg/mL solution B was used as a study control. Each mixture was then diluted by a further factor of two using solution B. The OD_560_ was then analyzed using the Epoch plate reader for each plate (OD_560_0_) [35]. Once this dark condition was measured, the plate was light treated according to one of the five treatments provided in Table 1, similar to the procedure for the singlet oxygen assay. After each light treatment, the plate was stored under aluminum foil for 5 min before reading OD_560_t_. The percentage increase in OD_560_ (Equation (3)) corresponded to the amount of superoxide anion produced (n = 9):

% OD_560_ Decrease = [OD_560_t_ − OD_560_0_]/OD_560_0_ × 100% (3)

Acetonitrile, imidazole, and RNO were purchased from Fisher Scientific (Hampton, NH, USA), while NBT, sodium phosphate monobasic monohydrate, and sodium phosphate dibasic heptahydrate were purchased from Sigma Aldrich (St. Louis, MO, USA).

### 2.4. Bacterial Strain and Growth Conditions

Stock cultures of standard strains of *S. mutans* (ATCC 33535) and *C. albicans* (ATCC 90028) from the American Type Culture Collection (ATCC; Rockville, MD, USA) were maintained at −80 °C, reactivated on CDC Anaerobe 5% Sheep Blood Agar plates (BBL, Becton, Dickinson and Company, Sparks, MD, USA), and incubated at 37 °C (5% CO_2_) (Isotemp CO_2_ incubator, Thermo Fisher Scientific, Marietta, OH, USA) for 48 h. Next, the cells were individually reactivated by transferring single colonies (10–12 for *S. mutans* and 5–8 for *C. albicans*) in 5 mL of culture medium and storing overnight in an incubator (5% CO_2_, 37 °C). In preparation for the MIC and MBC/MFC assays, single colonies of *S. mutans* were transferred into brain–heart infusion broth culture medium (“BHI”; BD BBL, Becton, Dickinson and Company, Sparks, MD, USA) with 1% glucose (also known as dextrose; Sigma Aldrich, St. Louis, MO, USA), while those of *C. albicans* were transferred into Difco^TM^ BD Trypticasein Soy Broth (“TSB”; Becton, Dickinson and Company, Sparks, MD, USA) with 0.5% yeast extract (Difco, FL, USA) and 1% glucose. Both cultures were then stored in an incubator (5% CO_2_, 37 °C) overnight (24 h for *S. mutans* and 20 h for *C. albicans*). Inocula were started from an absorbance of 0.08–0.10, read at an optical density (OD) of 600 nm for *S. mutans* and 0.25 at an OD of 540 nm for *C. albicans,* using an Epoch microplate reader (BioTek, Winnoski, VT, USA), each corresponding to 1.5 × 10^8^ colony forming units (CFU)/mL.

### 2.5. Minimum Inhibitory Concentration (MIC) Following aPDT

An aqueous curcumin solution was first prepared at a concentration of 2500 µg/mL in sterile ultrapure water. This concentration was then serially diluted with sterile ultrapure water by a factor of two in a 96-well plate (final concentration of 1250 µg/mL to 1.22 µg/mL). To these serially diluted curcumin solutions, an equal volume of 1 × 10^6^ CFU/mL of *S. mutans* in BHI or *C. albicans* in TSB was added. The final curcumin concentrations then ranged from 625 µg/mL to 0.61 µg/mL. The plates were then subjected to a light treatment (Table 1) of 56 mW/cm^2^ from royal blue LEDs (λ_max_ = 440–460 nm) using “BioLight”. After 20 h in an incubator (37 °C, 5% CO_2_; Isotemp CO_2_ incubator, Thermo Fisher Scientific, Marietta, OH, USA), the OD_600_ (for *S. mutans*) or the OD_540_ (for *C. albicans*) was read using the Epoch microplate reader spectrophotometer. The MIC was defined as the lowest concentration of curcumin in aqueous solution capable of inhibiting more than 99% of visible microorganism growth. A 1:1 *v*/*v* mixture of ultrapure water and BHI (lacking curcumin) served as a study control. For this assay, three runs were conducted, each in triplicate (n = 9).

### 2.6. Minimum Bactericidal or Fungicidal Concentration (MBC or MFC) Following aPDT

Immediately following completion of the MIC assay, 10 µL of each of the seven highest concentrations from the MIC test was plated on respective agar plates—CDC Anaerobe 5% Sheep Blood Agar plates (BBL, Becton, Dickinson and Company, Sparks, MD, USA) for *S. mutans* and Difco^TM^ BD Sabouraud Dextrose agar (Becton, Dickinson and Company, Sparks, MD, USA) for *C. albicans;* all plates were incubated for 24 h. The MBC (*S. mutans*) and MFC (*C. albicans*) were defined as the lowest concentration of curcumin in aqueous solution capable of killing the respective microorganism. The 1:1 *v*/*v* mixture of ultrapure water and BHI (lacking curcumin) served as a study control in these assays as well.

### 2.7. Statistical Analysis

All statistical analyses were conducted using IBM SPSS Statistics for Windows, Version 28.0 software (IBM Corp., Armonk, New York, NY, USA, source: https://www.ibm.com/products/spss-statistics (accessed on 30 July 2022)). The data were analyzed using a multi-factor univariate general linear model and post hoc Tukey tests (α = 0.05). The data are presented in the included bar plots as the mean ± one standard deviation. A detected statistical significance was accepted as *p* < 0.05.

## 3. Results

### 3.1. Confirming Curcumin Powder Structure and Chemistry

**Figure 1:**
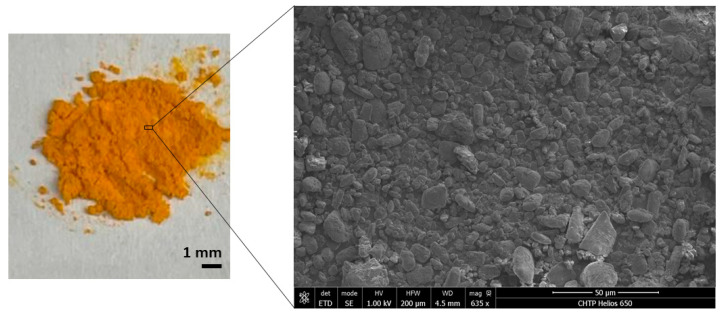
(**left**) iPhone image with 1 mm scale bar, and (**right**) FIB-SEM image of curcumin powder at a working distance of 4.5 mm and a scale bar of 50 µm.

Both the SEM and ATR-FTIR analyses confirmed the structure and chemistry of the curcumin powder investigated in this study. For example, SEM revealed that the curcumin powder was not uniform in shape or size, with most particles a few µm to 100 µm in size (Figure 1).

**Figure 2:**
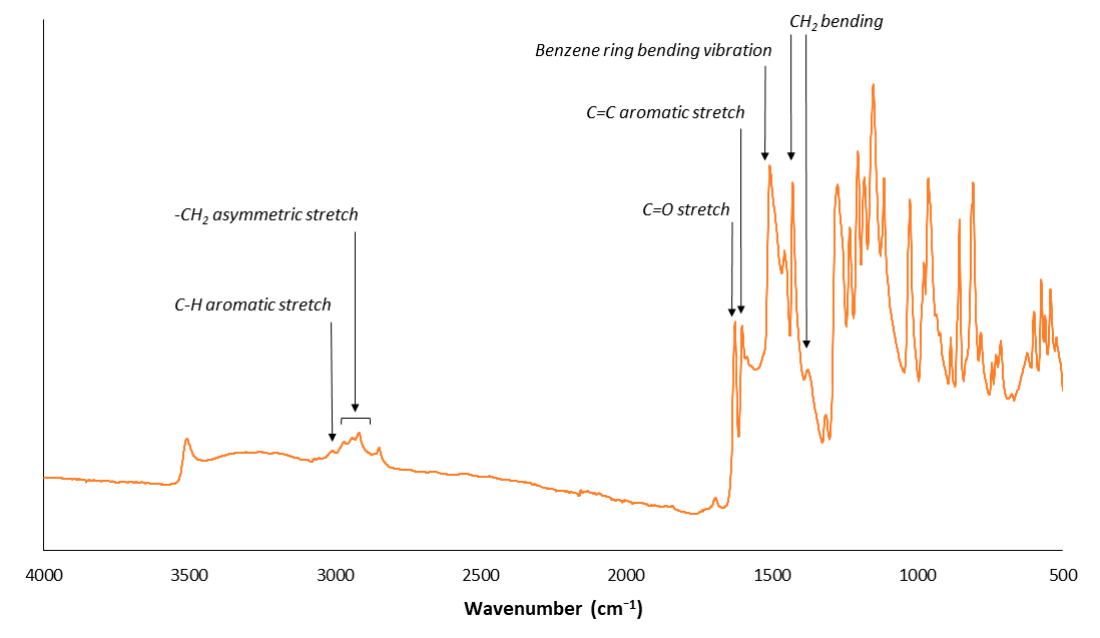
ATR-FTIR spectra of dry curcumin powder with some of the key functional groups identified.

Meanwhile, the ATR-FTIR analysis confirmed the presence of key functional groups of curcumin (Figure 2).

### 3.2. Light Absorbance by Curcumin

**Figure 3:**
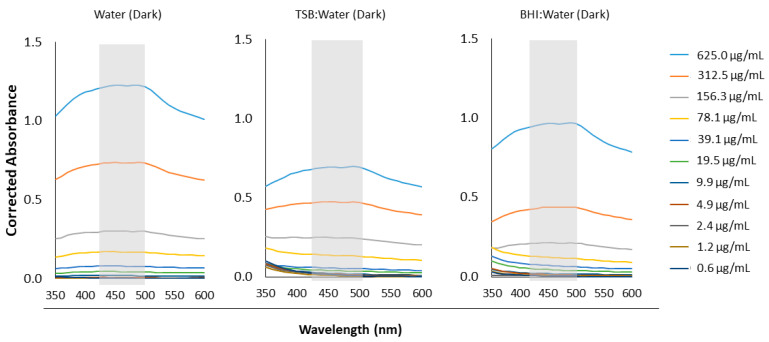
Absorbance of aqueous curcumin solutions as a function of curcumin concentration and media choice. Here, media choice was solely water (**left**), a 1:1 *v*/*v* mix of TSB and water (**middle**), and a 1:1 *v*/*v* mix of BHI and water (**right**). The broad peak range is indicated on each absorbance plot with a grey-shaded box.

Recognizing that our in vitro investigation of curcumin required the use of different media—aside from solely water—the absorbance of curcumin at different concentrations in relevant media was also reported here (Figure 3). The absorbance of the aqueous curcumin was notably impacted by the media choice when the curcumin concentration was greater than 156.3 µg/mL. At these higher concentrations (312.5 and 625.0 µg/mL), the light absorbance of curcumin was greater in water than it was in either the TSB or BHI media at matching wavelengths.

**Figure 4:**
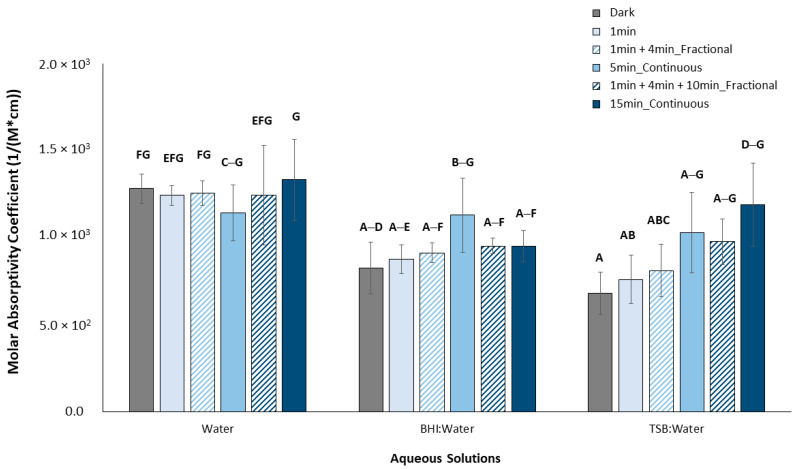
Molar absorptivity coefficients of aqueous curcumin solutions as a function of media and light treatment. Means that are statistically different are assigned different letters (*p* < 0.05, n = 5).

Next, the molar absorptivity coefficient of the aqueous curcumin solutions was found to be detectably impacted by media type (*p* < 0.001), total light time (*p* < 0.001), and light operation mode (*p* = 0.015) (Figure 4). The molar absorptivity coefficient is a measure of how strongly a specific chemical species absorbs light at a given wavelength.

The addition of either BHI or TSB media to the curcumin aqueous solution under dark conditions detectably reduced the molar absorptivity coefficient compared to curcumin in water only (*p* = 0.003 and *p* < 0.001, for each media, respectively). A reduction was also observed following the application of 1 min of light to either the BHI or TSB media samples compared to curcumin in water only (*p* = 0.045 and 0.003, respectively). The application of light for 5 min to curcumin in TSB in fractional mode also detectably reduced the molar absorptivity coefficient compared to curcumin in water only (*p* = 0.005)

### 3.3. ROS Release Following aPDT

**Figure 5:**
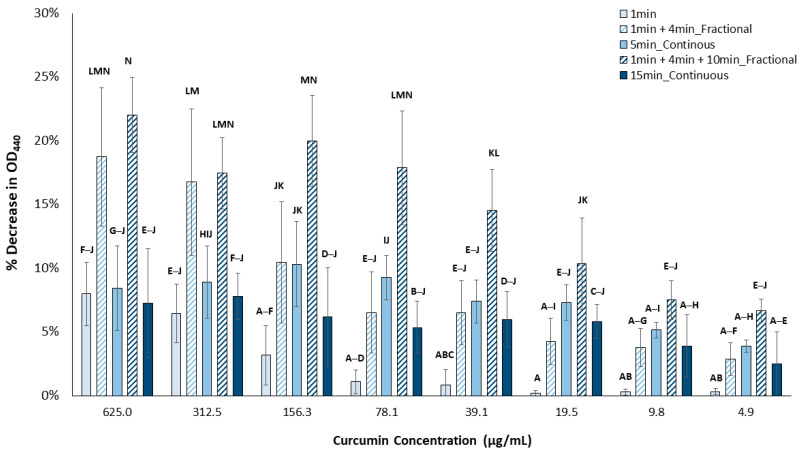
Singlet oxygen release following aPDT of aqueous curcumin solutions as a function of concentration and light treatment. Means that are statistically different are assigned different letters (*p* < 0.05, n = 9).

The production of singlet oxygen (Figure 5), as shown by a decrease in OD_440_, was significantly impacted by curcumin concentration, total time of light, and mode of light application (continuous or fractional) (*p* < 0.001 for all).

At curcumin concentrations greater than 19.5 µg/mL, the application of 5 min of fractional light produced detectably more singlet oxygen than 1 min (*p* < 0.02). For the 15 min fractional light treatment, all curcumin concentrations produced detectably more singlet oxygen than 1 min (*p* < 0.001). An increase in singlet oxygen output as light treatment time increased was also observed for a smaller range of curcumin concentrations (19–156 µg/mL with 5 min, and 19–39 µg/mL for 15 min) following continuous blue light energy (*p* < 0.001 for 5 min and <0.042 for 15 min). In addition, 5 min of fractional light resulted in detectably more singlet oxygen produced than 5 min of continuous blue light energy at 312.5 and 625 µg/mL only (*p* < 0.001 for both), but there were no differences at the other concentrations for the same light treatment. Meanwhile, 15 min of fractional blue light energy produced detectably more singlet oxygen than 15 min of continuous light for 39–625 µg/mL curcumin (*p* < 0.001 for all). Furthermore, there was an overall decrease in singlet oxygen production as the curcumin concentration decreased following each light treatment.

**Figure 6:**
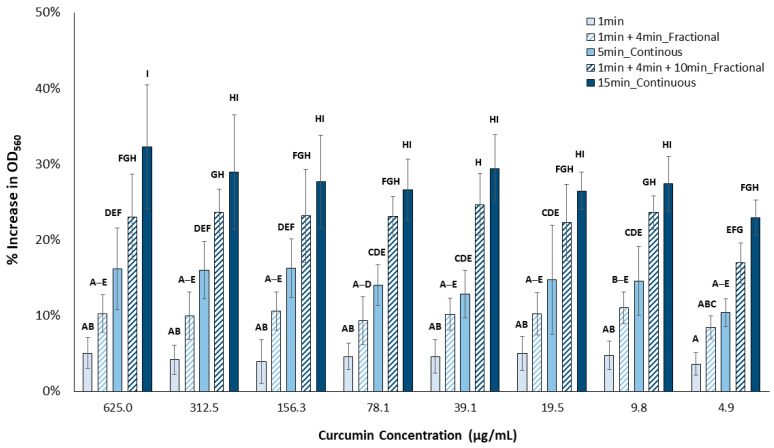
Superoxide anion release following aPDT of aqueous curcumin solutions as a function of concentration and light treatment. Means that are statistically different are assigned different letters (*p* < 0.05, n = 9).

The production of superoxide anions (or OD_560_ increase) was significantly impacted by curcumin concentration, total time of light, and mode of light application (continuous or fractional) (*p* < 0.001 for all) (Figure 6).

The application of fractional light for 15 min detectably increased the superoxide anion produced compared to 1 min only (*p* < 0.001 at each curcumin concentration), while applying only 5 min of fractional light did not produce a significant change compared to the 1 min treatment (*p* > 0.05). Meanwhile, increasing the amount of continuous light energy resulted in a detectably greater superoxide anion production for the 5 min (*p* < 0.006) and 15 min (*p* < 0.001) treatments, compared to the 1 min treatment. In addition, contrary to the singlet oxygen production results, there were no detectable differences in superoxide anion production between 5 min of fractional or continuous light energy at any curcumin concentration. Furthermore, only at 625 µg/mL was there detectably more superoxide anions produced following 15 min of continuous light compared to a similar amount of time of fractional blue light energy (*p* < 0.001).

### 3.4. Minimum Bactericidal or Fungicidal Concentration (MBC or MFC) Following aPDT

The dependence of MIC, MBC, and MFC on type of microorganism, curcumin concentration, and blue light energy was very evident in the results (Table 2).

*C. albicans* required 15 min of continuous or fractional blue light energy to determine the MIC, although the corresponding MFC was notably higher under the fractional mode than the continuous mode. Meanwhile, only 1 min of continuous or fractional blue light energy was necessary to observe an MIC and MBC for *S. mutans*. Lastly, increasing the amount of light energy reduced both the MIC and MBC for *S. mutans*, particularly under the fractional light mode.

## 4. Discussion

Curcumin has been previously investigated as a PS to mitigate cariogenic microorganisms through PDT [16,17,18,19,27,36], mainly in direct contact with the cells in planktonic cultures. However, the parameters utilized in aPDT investigations with curcumin as the photosensitizer are not only inconsistent, but there has been very little investigation of the photochemical reaction and ROS production necessary to generate the desired antimicrobial effect. Therefore, this study sought to provide fundamental evidence as to some of the parameters which impact the efficacy of curcumin as a photosensitizer in aPDT.

In this study, a broad peak for curcumin from at least 350 nm to 550 nm with no dependence of the shape on water-only or other media type (BHI or TSB) observed. This peak overlaps with the known peak wavelength of our blue LED light device (BioLight) operating at 410–500 nm and suggests that the pairing of blue light with curcumin should prove appropriate for aPDT investigations. It also matches the absorption peak of curcumin previously observed in different media [16,37,38]. The magnitude of the absorbance at 420 nm did show a dependence on media type and decreased in the order of water > BHI > TSB at curcumin concentrations of 156.3 µg/mL and higher. Furthermore, the molar absorptivity of curcumin also showed some dependence on media type, with dark and pre-treatment with one minute of blue light resulting in a lower curcumin molar absorptivity coefficient in BHI or TSB compared to in water alone. It is possible that under such conditions, the ingredients of either culture media interfered in light passage and absorbance by the curcumin particles, as the media might have absorbed some of the light itself. It is also possible that above a certain concentration (here shown to be at least 156.3 µg/mL), the curcumin solubility was influenced by the media, and differences in optical quenching by excessive dye was more apparent. In a previous study evaluating curcumin at a maximum concentration of ~3 µg/mL with 24, 48, or 74 J/cm^2^ blue LED light application, Paschoal et al. (2013) also suggested that optical quenching was responsible for the lack of correlation between curcumin concentration and the photokilling rate [16]. In addition, Erez et al. (2014) showed a dependence of curcumin light absorbance on the pH of the media, with neutral solutions showing a higher absorbance. Thus, they partially attributed this dependence to the protonation of curcumin [38]. It is possible then that other ingredients in the BHI and TSB media may compete for this proton interaction with curcumin, particularly when the curcumin concentration is higher. In our study, additional application of blue light energy (i.e., beyond 1 min) overcame any dependence on media choice. This could be the result of some photodegradation of the curcumin or other ingredients in the culture media such as glucose [39], and a decrease in excessive dye concentration following the application of a greater amount of blue light energy. Overall, the first general hypothesis stating that the absorption of light by curcumin would be affected by media choice was supported. This study provides the first evidence of the potential impact of culture media selection on any benchtop evaluation of aPDT, a study design variable that was previously overlooked.

In this study, a strong dependence of singlet oxygen on curcumin concentration and mode of light application was observed. However, this dependence was much less apparent with superoxide anion production. In fact, at higher concentrations of curcumin, there was notably more singlet oxygen produced following fractional light treatment than continuous light treatment. In photochemistry, there are two main types of reactions: type I and type II. In the type I pathway, electron transfer and/or hydrogen abstraction between the PS and substrates generate free radicals, which rapidly react with molecules such as water or oxygen to produce superoxide, hydrogen peroxide, and hydroxyl radicals [14,30]. In a type II reaction involving energy transfer to oxygen, singlet oxygen (^1^O_2_) is produced [30]. While the type I and II processes generally occur in parallel during aPDT [14], the PS activation towards the success of either is very dependent on the environment [40,41,42,43,44]. For example, curcumin is known to show some degradation in response to excessive heat [45] and blue light irradiation [46], forming by-products such as vanillin. While vanillin has been shown to exhibit some cytotoxic behavior against microbes [47], there is also evidence in the literature that vanillin particularly favors scavenging singlet oxygen, although other ROS may also be at risk of scavenging to a lesser degree [48]. As a result, with continuous light mode producing slightly more heat within the curcumin solution, it is likely that more curcumin degradation and singlet oxygen scavenging occurred than would with the fractional light mode; thus, the less singlet oxygen detected in the continuous mode is due to greater scavenging. Future studies will investigate how the production of the degradation by-products, such as vanillin, contribute to the efficacy of the aPDT approach.

In a healthy cell, moderate levels of ROS support normal cellular proliferation and homeostasis [49]. An antioxidant system found in cells, including enzymes such as superoxide dismutase and non-enzymatic chemicals such as vitamin C, is responsible for regulating the ROS concentration [49,50]. However, if the healthy balance of ROS is disrupted—as occurs during aPDT with intentional ROS generation—cellular constituents become damaged. In this study, more energy was necessary to inhibit and kill *C. albicans* compared to *S. mutans*. This study provides evidence supporting the second general hypothesis that blue light energy would impact the MIC and MBC or MFC of the cells investigated. As the first study to confirm the cell-specific dependence of MIC and MBC or MFC on blue light energy, future studies will be able to use the knowledge gained here to optimize the energy needs for efficacious aPDT.

In addition, the continuous light mode resulted in a lower MIC and MFC for *C. albicans* after 15 min of total blue light energy than those observed for the fractional mode. The observations for *C. albicans* match more closely with the superoxide anion generation trend, with the highest ROS generation after 15 min of continuous light. In the literature, it has been reported that the ROS generated from the type I mechanism is generally more toxic than the singlet oxygen generated in the type II pathway [14,51]. Singlet oxygen, while highly reactive, has a very short lifetime and has been reported in the range of 10–200 ns [52,53,54], depending on the analytical test and media used. In addition, singlet oxygen can only diffuse to an upwards limit of ~220 nm in cells in the absence of any significant interaction with biomolecules [52,55,56]. It is also possible that the different response of the two microorganisms studied here may be due to a greater initial cell surface area (at matching CFU/mL) for *C. albicans* compared to *S. mutans.* While *C. albicans* are larger cells at 5–6 µm [57], *S. mutans* have an average cell size of 0.5–0.75 µm [58]. The lack of dependence of the MIC and MBC on the light application mode for *S. mutans* is likely due in part to the reduced molar absorptivity of curcumin in BHI. However, there was a noted dependence on light mode at higher blue light energies. The MIC and MBC of *S. mutans* following 5 or 15 min of fractional light were lower than the matching total time using the continuous mode. This mode dependence is contrary to that observed for *C. albicans* and could indicate that aPDT targeting of *S. mutans* is more dependent on singlet oxygen production, as the continuous mode likely results in greater thermal degradation of curcumin to form vanillin, a degradation by-product and singlet oxygen scavenger [48]. As a result, this study provided evidence supporting the third general hypothesis that the light mode would impact the MIC, MBC, or MFC; there was a noted dependence on cell type for which light mode had a stronger aPDT response. This is the first study to systematically investigate the impact of the light mode on aPDT responses and such knowledge will also be greatly beneficial in future curcumin-mediated aPDT. Furthermore, the likely greater dependence of *S. mutans* inhibition on singlet oxygen production (type II photochemistry) than superoxide anion production has also been reported following exposure to other PSs [59], but not curcumin to our knowledge. As *S. mutans* are smaller cells, the limited diffusion depth for singlet oxygen may then not be as great an issue as with *C. albicans*. Furthermore, *S. mutans* showed an overall dependence on blue light energy, with increased energy resulting in lower MIC and MBC values.

The passage of a PS into a microbiological cell is not a passive process, as the cell membrane acts as a selective barrier to free diffusion [30]. The penetration of a PS molecule depends on its size, charge, and relative solubility in water and lipids, amongst other properties [13,40,60]. It is traditionally thought that only cells with the necessary accumulation of the PS are killed during light exposure in aPDT. Curcumin has been reported to be transported across the cell membrane of various microbial cells, including *S. mutans*, albeit largely when dissolved in organic solvents [61]. Interestingly, curcumin at the cell surface has also been shown to damage the cell membrane of microbial cells due to membrane leakage [62]; as such, its poor aqueous solubility may not be as much of a limiting factor as often thought in aPDT. For example, in our study the undissolved curcumin powder was a few hundred micrometers in size and, while notably larger than the size of the microbial cells investigated, a concentration-dependent antimicrobial response was still observed. Note that in this study, a pre-irradiation time of 5 min was set for ROS quantification, as well as for MIC and MBC/MFC detection. Future investigations will consider how longer pre-irradiation times may improve the curcumin-mediated aPDT response of cariogenic microorganisms in media. As greater blue light energy did produce more ROS and these results supported the fourth hypothesis, future studies will seek to optimize the requisite energy necessary to manage a healthy microbiota. This is the first study to systematically investigate ROS production as a function of different blue energy levels and the findings will greatly help in the design of future aPDT-based material systems.

## 5. Conclusions

In conclusion, this study further confirmed the potential for using curcumin as a photosensitizer in royal blue LED-activated aPDT involving both *S. mutans* and *C. albicans*. The synergistic investigation of curcumin absorbance, ROS production, and antimicrobial response provided new evidence on the impact of two crucial light parameters—energy and mode—on curcumin efficacy as a PS. Firstly, the light absorbance of curcumin showed some dependence on the media at higher curcumin concentrations. In addition, as the blue light energy increased so did the production of ROS and the resultant MIC of either species was reduced. Furthermore, the dependence on the light mode of the MIC, MBC, or MFC was directly linked to species with *S. mutans* being more sensitive to continuous delivery than *C. albicans*. Future investigations will seek to apply the knowledge gained from this study towards the development of a curcumin-loaded formulation intended for dental disease management involving oral pathogens.

## Figures and Tables

**Table 1:** Light treatments.

No.	Mode	Segment 1	Segment 2	Segment 3	Segment 4	Segment 5
1	Continuous	1 min on	-	-	-	-
2	Fractional	1 min on	5 min off	4 min on	-	-
3	Continuous	5 min on	-	-	-	-
4	Fractional	1 min on	5 min off	4 min on	5 min off	10 min on
5	Continuous	15 min on	-	-	-	-

**Table 2:** Minimum inhibitory concentrations (MICs) and bactericidal/fungicidal concentrations (MBCs/MFCs) of aqueous curcumin as a function of light treatment.

		Light Treatment (Royal Blue LED, 56 mW/cm^2^)				
Species	Test	Mode	0 min	1 min	5 min	15 min
*C. albicans*	MIC	Continuous	ND	ND	ND	9.8 µg/mL
		Fractional			ND	19.5 µg/mL
	MFC	Continuous	ND	ND	ND	9.8 µg/mL
		Fractional			ND	625 µg/mL
*S. mutans*	MIC	Continuous	ND	39.1 µg/mL	39.1 µg/mL	19.5 µg/mL
		Fractional			19.5 µg/mL	4.9 µg/mL
	MBC	Continuous	ND	156.3 µg/mL	78.1 µg/mL	39.1 µg/mL
		Fractional			39.1 µg/mL	9.8 µg/mL

ND indicates that the minimum concentration was not detected.

## Data Availability

The data presented in this study are available on request from the corresponding author.
